# Presbyterian Hospital Report

**Published:** 1896-08

**Authors:** 


Presbyterian Hospital Report. Medical and Surgical Report of the Presbyterian Hospital in the City of New York. Volume I., January, 1896. Edited by Andrew J. McCosh, M. D., and Walter B. James, M. D. New York: The Knickerbocker Press.

This report embraces some of the work done at the Presbyterian hospital and is the forerunner of contemplated annual reports which shall review some of the interesting cases received at this hospital.

After giving the names of the managers, medical board, visiting and attending staffs, twenty-six cases of rare diseases are reported, such as caisson disease, tetany, extra-uterine gestation, foreign body in stomach, tumors of the kidney, coccygeal cyst, and the like. Then follows the statistics of the medical and surgical wards, list of operations and the formulæ of the operating pavilion.

This report is worthy of emulation by other hospital boards and is in the line of the work mapped out by the former Erie county hospital staff. W. C. K.

